# Immediate and Sustained Effects of Song‐Based Language Learning on English as a Foreign Language Learners’ Academic Development, Cognitive Engagement, and Motivation

**DOI:** 10.1002/brb3.71532

**Published:** 2026-06-08

**Authors:** Yanyi Wang, Morteza Jabrealy, Siros Izadpanah

**Affiliations:** ^1^ International Cooperation Division Shanghai Urban Construction Vocational College Shanghai China; ^2^ Department of English Language Teaching, Zanjan Branch Islamic Azad University Zanjan Iran

**Keywords:** academic development, cognitive engagement, English as a foreign language (EFL), motivation, learning through song

## Abstract

**Background:**

This study examined the immediate and sustained effects of song‐based language learning on English as a foreign language (EFL) learners’ academic development, cognitive engagement, and motivation for learning. The study is primarily grounded in self‐determination theory and sociocultural theory and is additionally informed by the input and output hypotheses and multimodal learning theory.

**Method:**

A quasi‐experimental design was employed with 200 elementary‐level EFL learners drawn from eight private language institutes in Urmia, Iran. Participants were assigned to experimental and control groups. The experimental group received 11 weeks of song‐based English instruction, while the control group received traditional instruction. Data were collected at three time points—pretest, immediate posttest, and follow‐up—using validated questionnaires measuring academic development, cognitive engagement, and motivation for learning. Repeated‐measures analysis of variance (ANOVA) was used to analyze the data.

**Results:**

The results revealed that learners exposed to song‐based instruction demonstrated significantly greater gains in academic development, cognitive engagement, and motivation for learning at the immediate posttest compared to those in the control group. Importantly, these gains were largely maintained at the follow‐up stage, indicating that the positive effects of song‐based instruction were sustained over time.

**Conclusion:**

The findings suggest that integrating songs into EFL instruction can promote not only immediate learning benefits but also enduring improvements in learners’ academic development, cognitive engagement, and motivation. The study contributes to the growing body of research on arts‐integrated pedagogies in language education and offers important pedagogical implications for EFL teachers and curriculum designers seeking to foster sustained learner engagement and development.

## Graphical abstract

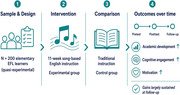

**Abbreviations**
EFL: English as a foreign language

## 1 Introduction

English as a foreign language (EFL) instruction has traditionally emphasized explicit grammar teaching, vocabulary memorization, and repetitive practice, particularly in contexts where learners have limited opportunities to use English outside the classroom (Richards and Rodgers 2014; Pawlak 2021; Şahinkaya 2024). While such approaches continue to shape classroom practice in many educational systems, their effectiveness has increasingly been questioned in applied linguistics research. Evidence suggests that form‐focused instruction alone may be insufficient to sustain learners’ cognitive engagement, motivation, or long‐term academic development (Dörnyei 2005; Shabani and Gholami 2020).

In exam‐oriented contexts, these instructional practices have also been associated with heightened anxiety and reduced willingness to communicate, both of which may constrain effective language learning (Dörnyei and Ryan 2015; Dörnyei and Ushioda 2013). These limitations have led researchers to call for pedagogical approaches that address not only linguistic accuracy but also the cognitive and affective conditions that shape language learning processes (Tang 2025). Within this broader shift, the use of songs and music in EFL instruction has attracted sustained scholarly attention (Agustina et al. 2025). Research suggests that music‐based instruction may support listening comprehension, pronunciation, and vocabulary development through rhythm, melody, and repetition, which facilitate phonological processing and memory consolidation (Besson et al. 2011; Schön et al. 2004; Urbaite 2025).

Songs are also considered emotionally engaging forms of input that may lower learners’ affective filters (Krashen 1985; Engh 2013). These characteristics align with multimodal learning theory, which proposes that learning is enhanced through the integration of auditory and verbal channels (Mayer 2009). Neurocognitive research further indicates that musical input activates neural systems associated with attention, working memory, and language processing (Patel 2011; Moreno et al. 2011). Despite these promising findings, recent evidence syntheses urge caution in how confidently song‐based instruction is discussed in the field. A large‐scale systematic review published in leading journals reported that, although many studies have shown positive effects of songs on language‐related outcomes, a considerable number of intervention studies lack the methodological strength necessary to draw firm causal conclusions (Hamilton et al. 2024). This review particularly indicates that a large number of studies assess outcomes only through immediate posttests, which makes it difficult to determine whether the reported benefits persist after the instructional period has ended. This underscores the need for methodologically robust classroom research that examines not only short‐term gains but also the sustainability of learning outcomes over time.

In addition to cognitive and linguistic outcomes, music‐based instruction has been linked to learner motivation and engagement. Drawing on self‐determination theory (SDT), prior research suggests that musical activities may support learners’ psychological needs for autonomy, competence, and relatedness, thereby fostering more self‐determined forms of motivation (Noels et al. 2000; Ryan and Deci 2020). Recent studies in prestigious journals such as *System* further emphasize motivation and engagement as central mechanisms underlying language learning, reinforcing the importance of treating these constructs as primary outcomes rather than peripheral byproducts (Lei and Ye 2025; Zou et al. 2025). Shared musical experiences may also promote classroom cohesion and agentic engagement, which in turn can enhance learners’ willingness to participate and persist in language learning tasks (Reeve and Shin 2020; Xu and Li 2025). These affective dimensions are particularly salient in EFL settings, where learners often report limited confidence and restricted opportunities for meaningful language use (Dörnyei and Ushioda 2013; Lamb 2017).

Nevertheless, several limitations in the existing literature remain evident. Many studies employ short intervention periods or small samples, limiting the generalizability of findings to formal instructional contexts (Yang and Li 2025). Moreover, prior research has frequently examined isolated outcomes such as vocabulary acquisition or pronunciation accuracy rather than investigating how song‐based instruction simultaneously influences academic development, cognitive engagement, and motivation within an integrated empirical framework (Dincer and Yesilyurt 2021). To ensure theoretical coherence, the current study primarily draws on SDT to explain learners’ motivational processes and sociocultural theory (SCT) to frame the social‐interactional and co‐constructive nature of song‐based learning. Supporting ideas from multimodal learning theory and the input/output hypotheses are referenced as complementary perspectives that conceptually enrich, rather than compete with, the central SDT‐ and SCT‐driven rationale.

This narrowing of focus provides a more unified interpretation of how song‐based instruction can foster both autonomous motivation and socially mediated cognitive engagement in EFL contexts such as Iran, where grammar‐focused instruction remains prevalent and alternative multimodal methods are underexplored (Pishghadam et al. 2013). Furthermore, few studies in these settings have incorporated follow‐up measurements, leaving unanswered questions regarding the durability of instructional effects.

In response to these gaps, the present study examines the immediate and sustained effects of learning English through songs on EFL learners’ academic development, cognitive engagement, and motivation for learning within a formal instructional context. Employing a quasi‐experimental design with pretest, posttest, and follow‐up assessments, the study compares learners receiving song‐based instruction with those taught through traditional methods, in line with methodological recommendations for classroom‐based applied linguistics research (Mackey and Gass 2021; Plonsky and Oswald 2017). This study moves beyond the common rationale that music is engaging or enjoyable by showing how song‐based instruction may shape multiple dimensions of EFL learning and whether these effects endure beyond immediate instruction, offering new evidence from an underrepresented instructional context. In this study, academic development refers to learners’ perceived progress in core language skills, including vocabulary growth, comprehension, and linguistic accuracy (Wells 2010). Cognitive engagement is conceptualized as learners’ sustained attention, mental effort, retention, and active participation during learning activities (Fredricks et al. 2004; Reeve 2013). Motivation for learning is defined as learners’ interest, enjoyment, persistence, and attitudes toward learning English, encompassing both intrinsic and extrinsic dimensions (Ryan and Deci 2020; Dörnyei and Ushioda 2013).

The study is informed by a focused theoretical framework in which SDT and SCT serve as the primary explanatory lenses, while multimodal learning theory and the input and output hypotheses are treated as complementary perspectives that clarify the nature of song‐based input and classroom interaction (Mayer 2009; Krashen 1985). From an SDT perspective, song‐based instruction is expected to support autonomy through enjoyable, low‐pressure activities; competence through scaffolded exposure to comprehensible language; and relatedness through shared musical experiences (Ryan and Deci 2020). SCT conceptualizes classroom work with songs as socially mediated activity involving scaffolding, negotiation of meaning, and collaborative interpretation (Vygotsky 1978). Within this focused framework, song‐based instruction is understood as a multimodal pedagogical approach that facilitates comprehensible input, meaningful output, social interaction, and support for psychological needs—conditions conducive to enhanced motivation, deeper cognitive engagement, and sustained academic development. By addressing both methodological and contextual limitations in prior research, the present study contributes to ongoing discussions in applied linguistics concerning the role of arts‐integrated pedagogy in EFL classrooms and the sustainability of instructional effects over time (Loewen and Plonsky 2021).

## 2 Literature Review

### 2.1 Learning Language Through Songs in EFL Instruction

In recent decades, research in second and foreign language education has increasingly questioned exclusively form‐focused instructional approaches, particularly in contexts with limited exposure to the target language (Hamilton et al. 2024). Consequently, scholars have explored pedagogical practices that integrate cognitive, affective, and social dimensions of learning. Among these, songs have received attention as a potentially integrative instructional resource (Fonseca‐Mora et al. 2011). Songs are commonly conceptualized as pedagogical tools that combine linguistic input with rhythm and repetition (Besson et al. 2011; Schön et al. 2004). From a pedagogical perspective, they provide authentic language input and may increase learners’ receptivity to linguistic forms (Patel 2011; Moreno et al. 2011). Despite growing empirical support, song‐based instruction has often been treated as a supplementary or motivational activity rather than as a systematically designed instructional approach. This limited conceptualization has constrained the depth of empirical investigation into how songs function as a core pedagogical resource in EFL classrooms, highlighting the need for research that examines their broader academic and psychological effects. Nevertheless, most prior studies have been limited by short duration, small samples, and a narrow focus on immediate language gains, leaving open the question of whether song‐based instruction produces sustained and academically meaningful effects.

### 2.2 Songs and Academic Development in EFL

Academic development in language learning extends beyond discrete test scores to cover learners’ overall progress in comprehension, accuracy, vocabulary growth, and effective language use over time (Huang and Shih 2011; Liu 2025). While many studies examining songs in EFL contexts report positive effects on specific linguistic skills particularly vocabulary acquisition, pronunciation, and listening comprehension, these outcomes are often investigated in isolation and over short instructional periods.

The repetitive and rhythmic nature of songs has been shown to support lexical retention and phonological awareness, which are foundational components of academic development in language learning. Moreover, emotionally engaging input has been associated with deeper processing and longer‐term retention, suggesting that songs may contribute to sustained academic growth rather than short‐term performance gains. However, systematic reviews caution that many existing studies lack rigorous experimental design, making it difficult to draw firm conclusions about the impact of songs on learners’ broader academic development (Jiang 2024).

As a result, although songs appear to support specific language skills, their contribution to holistic academic development remains underexplored. Importantly, while songs have been associated with improved retention of lexical and phonological features, relatively few studies have examined whether such gains are maintained beyond the immediate instructional period, leaving the durability of academic development effects insufficiently understood. This gap underscores the need for research that conceptualizes academic development as a multidimensional construct within song‐based EFL instruction.

### 2.3 Songs and Cognitive Engagement

Cognitive engagement refers to learners’ sustained attention, mental effort, persistence, and strategic processing during learning activities (Lo 2025). Educational research consistently demonstrates that cognitive engagement plays a central role in determining learning quality and academic outcomes. However, within music‐based EFL research, cognitive engagement has rarely been treated as an explicit outcome variable.

Songs may promote cognitive engagement by directing attention through melody and rhythm and providing repeated exposure to linguistic input (Urbaite 2025). Multimodal learning theory provides a useful explanatory framework for this effect, suggesting that the integration of auditory and verbal channels may enhance attentional focus and memory formation (Mayer 2009). Prior neurocognitive research has proposed that musical input may be associated with greater neural activation related to attentional control and working memory; however, this interpretation remains speculative in the current study because such processes were not directly measured (Patel 2011). Despite these promising findings, the existing literature remains methodologically and conceptually limited in several respects. Many studies have focused on short‐term gains, small or context‐specific samples, and narrowly defined outcomes such as vocabulary recall or pronunciation accuracy. As a result, the durability of song‐based learning effects and their broader contribution to academic development remain insufficiently established. In addition, cognitive engagement is often inferred from surface‐level participation rather than measured directly, leaving the mechanisms underlying learning gains underexplored. Taken together, these studies suggest that songs may be pedagogically valuable; however, many of them rely on short interventions, small samples, or single outcome measures, making it difficult to determine whether observed benefits are durable, transferable, or academically meaningful beyond immediate classroom performance. Moreover, most studies emphasize learner enjoyment or participation rather than analytically examining cognitive engagement as a distinct construct.

### 2.4 Songs and Motivation for Learning

Motivation has long been recognized as a critical factor in developmental language learning (Song 2024). SDT offers a comprehensive framework for understanding how instructional practices influence learner motivation by addressing basic psychological needs for autonomy, competence, and relatedness (Ryan and Deci 2017, 2020). In EFL contexts, satisfaction of these needs has been associated with increased persistence, engagement, and achievement (Noels et al. 2000; Dörnyei and Ushioda 2013).

Song‐based instruction has been widely associated with enhanced learner motivation due to its capacity to reduce anxiety, increase enjoyment, and foster positive classroom dynamics (Zakiyah et al. 2024). Songs may support autonomy by allowing creative expression, competence by presenting language in manageable and memorable forms, and relatedness through shared musical experiences (Ushioda 2011; Vallejo and Pérez Ortega 2024). Empirical studies consistently report increased enjoyment and positive attitudes toward language learning when songs are integrated into instruction.

However, much of the existing research relies on short‐term self‐report measures, limiting insights into the sustainability of motivational effects and their relationship with cognitive engagement and academic development. This highlights the need for research that situates motivation within a broader explanatory model rather than treating it as an isolated affective outcome. Moreover, existing studies rarely investigate whether increases in motivation associated with song‐based instruction are sustained over time or how such motivational patterns interact with learners’ continued cognitive engagement and academic development.

Although the present study integrates multiple theoretical perspectives, these frameworks are not without tension. While SDT emphasizes individual psychological needs, sociocultural perspectives foreground socially mediated learning processes. The present findings suggest that song‐based instruction may function at the intersection of these perspectives, simultaneously supporting individual motivation and collective engagement.

### 2.5 Integrating Academic Development, Cognitive Engagement, and Motivation

Although academic development, cognitive engagement, and motivation are frequently examined independently, theoretical and empirical research suggests that these constructs are deeply interconnected. Motivation influences learners’ willingness to invest effort (Heo 2025). Cognitive engagement reflects the quality of that investment (Sulis and Mercer 2025), and academic development represents the cumulative outcome of sustained engagement over time (Namaziandost and Hwang 2025). From this perspective, song‐based instruction may function as a pedagogical catalyst that enhances motivation, which in turn increases cognitive engagement and ultimately supports academic development. Despite the plausibility of this integrated model, few empirical studies have examined these three constructs simultaneously within a unified theoretical and methodological framework. This fragmentation limits the field's ability to explain how and why songs influence learning outcomes in EFL contexts.

Across the literature, several recurring limitations are evident. Many studies employ short intervention periods, small sample sizes, or nonexperimental designs, which constrain generalizability. Additionally, research conducted in underrepresented EFL contexts, including Iran, remains limited, despite the dominance of traditional grammar‐focused instruction in such settings (Pishghadam et al. 2013). These gaps underscore the need for methodologically rigorous research that integrates multiple learner outcomes within diverse educational contexts. In particular, the scarcity of studies incorporating follow‐up (delayed) measurements limits current understanding of whether the cognitive and affective benefits of song‐based instruction persist after the intervention concludes. Addressing these gaps, the present study investigates the immediate and sustained effects of learning English through songs on EFL learners’ academic development, cognitive engagement, and motivation for learning. By adopting an integrated theoretical perspective and a quasi‐experimental design with pretest, posttest, and follow‐up measurements, the study seeks to examine not only whether song‐based instruction leads to improvement but also whether such effects are maintained over time within a formal instructional context.

The integrated theoretical framework of the present study is grounded primarily in SDT and SCT, and secondarily informed by multimodal learning theory and the input and output hypotheses. Within this framework, learning language through songs is conceptualized as a multimodal pedagogical intervention that supports learners’ psychological needs and socially mediated interaction, thereby influencing specific affective (motivation) and cognitive (perceived cognitive engagement) processes over time. The integrated theoretical framework of the present study is grounded primarily in SDT and SCT, and secondarily informed by multimodal learning theory and the input and output hypotheses. Learning language through songs is conceptualized as a multimodal pedagogical intervention that provides emotionally engaging input, opportunities for meaningful output, and socially mediated interaction. Importantly, the framework assumes that these relationships operate over time, such that increases in motivation and cognitive engagement resulting from song‐based instruction contribute to both immediate gains and the maintenance of academic development at a delayed stage.

**FIGURE 1:**
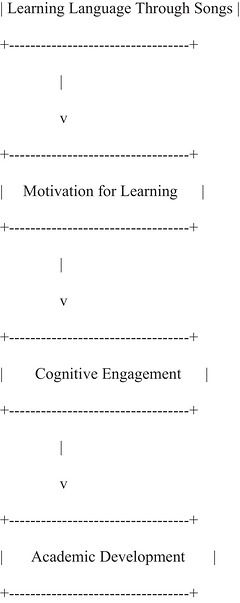
Integrated theoretical framework of the study.

This study is guided by an integrated theoretical framework that explains how learning language through songs influences learners’ academic development through motivational and cognitive processes. Within this framework, learning language through songs is hypothesized to have a direct positive effect on motivation for learning, as songs support learners’ psychological needs for autonomy, competence, and relatedness. Increased motivation is expected to enhance cognitive engagement, reflected in greater attention, mental effort, and persistence during learning activities. Sustained cognitive engagement, in turn, is hypothesized to contribute to academic development, manifested in learners’ overall progress in English language skills. Thus, motivation and cognitive engagement are conceptualized as key explanatory mechanisms through which song‐based instruction influences academic development. This perspective is consistent with SDT and SCT, which together highlight how supportive, socially mediated learning environments can enhance motivation, cognitive engagement, and academic development (Campbell et al. 1996). Within this framework, learning language through songs is conceptualized as a multimodal pedagogical intervention that supports learners’ psychological needs and socially mediated interaction, thereby influencing specific affective (motivation) and cognitive (perceived cognitive engagement) processes over time. The hypothesized relationships among song‐based instruction, motivation, cognitive engagement, and academic development are illustrated in Figure 1.

Direct and indirect pathways are assumed, with motivation and cognitive engagement functioning as theoretically explanatory mechanisms linking song‐based instruction to academic development. This study offers valuable contributions to both the practice and theory of EFL instruction. By highlighting the pedagogical potential of songs, it addresses current challenges in language learning and opens new pathways for research, instructional innovation, and learner engagement. Building on the integrated theoretical framework discussed above, the present study formulates several hypotheses regarding the effects of song‐based instruction on EFL learners’ academic development, cognitive engagement, and motivation. Drawing primarily on SDT and SCT, it is expected that song‐based learning activities can create an engaging and supportive learning environment that promotes learners’ participation and intrinsic motivation. In addition, insights from multimodal learning theory and the input and output hypotheses suggest that musical input combined with linguistic content may facilitate language processing and encourage deeper cognitive engagement. Accordingly, the following hypotheses are proposed:

**Hypothesis 1**. *Learners receiving song‐based instruction will report significantly greater gains in perceived academic development than learners receiving traditional instruction*.
**Hypothesis 2**. *Learners receiving song‐based instruction will exhibit higher levels of cognitive engagement than learners receiving traditional instruction*.
**Hypothesis 3**. *Learners receiving song‐based instruction will report higher motivation for learning than learners receiving traditional instruction*.

### 2.6 Research Questions

Guided by the integrated theoretical framework of the study, this research aims to examine the effects of learning English through songs on EFL learners’ academic development, cognitive engagement, and motivation for learning. In particular, the study investigates both the immediate and sustained impacts of song‐based instruction in comparison with traditional instructional approaches. Accordingly, the following research questions are proposed:

RQ1. Does learning English through songs lead to immediate and sustained improvements in EFL learners’ academic development compared with traditional instruction?
RQ2. Building on RQ1, does learning English through songs lead to immediate and sustained changes in EFL learners’ cognitive engagement during the learning process?
RQ3. Following RQ1 and RQ2, to what extent does song‐based instruction influence EFL learners’ immediate and sustained motivation for learning English?

## 3 Methodology

### 3.1 Research Design

The present study adopted a quasi‐experimental pretest–posttest–follow‐up design to examine the immediate and sustained effects of song‐based instruction on EFL learners’ academic development, cognitive engagement, and motivation for learning. This design was selected to allow for the examination of changes over time as well as differences between instructional conditions in a natural classroom setting, where random assignment at the individual level was not feasible (Creswell and Creswell 2018; Mackey and Gass 2021; Plonsky and Oswald 2017).

Two intact groups were assigned to an experimental condition and a control condition. The experimental group received song‐based instruction, while the control group received traditional instruction aligned with the existing curriculum. Both groups followed the same instructional objectives, content coverage, and instructional time, ensuring that any observed differences could be attributed to the instructional approach rather than to extraneous factors.

Importantly, the inclusion of a follow‐up (delayed) measurement allowed the study to examine not only whether song‐based instruction produced immediate gains, but also whether these effects were maintained beyond the instructional period. Incorporating delayed assessment is recommended in applied linguistics research to evaluate the durability of instructional effects and to distinguish short‐term performance gains from more sustained learning outcomes (Plonsky and Oswald 2017).

### 3.2 Participants and Sampling

The target population of the present study consisted of approximately 2000 elementary‐level EFL learners enrolled in eight private language institutes in Urmia, Iran. These institutes follow similar instructional curricula and assessment procedures, making them suitable for comparative classroom‐based research. To determine an adequate sample size, the Krejcie and Morgan (1970) sampling table was consulted. Based on the size of the target population, a minimum sample of 181 participants was required. To increase statistical power and to account for potential attrition during the intervention period, a total of 200 learners were recruited for the study. Because random assignment of individual learners was not feasible due to institutional and scheduling constraints, intact classes were used, consistent with standard practice in classroom‐based applied linguistics research. A multistage sampling procedure was employed. First, eight language institutes were selected based on accessibility and administrative consent. Within these institutes, intact elementary‐level classes were identified. These intact classes were then randomly assigned to either the experimental group or the control group, resulting in two groups of equal size (*n* = 100 per group).

Participants ranged in age from 14 to 20 years and included an equal number of male and female learners (50% male, 50% female). Although the age range was relatively broad, all participants were classified as elementary‐level learners based on institutional placement tests administered prior to enrollment, ensuring comparable language proficiency across groups. The average class size ranged from 10 to 15 learners, which is typical for language institute settings in Iran. Both groups were taught by the same instructor, followed the same syllabus, and received equal instructional time to control for teacher and curriculum effects.

All participants took part voluntarily. Informed consent was obtained from adult learners, and parental consent was secured for participants under the age of 18. Participants were informed of the purpose of the study, assured of the confidentiality of their responses, and notified of their right to withdraw from the study at any stage without penalty. All procedures adhered to established ethical guidelines for research involving human participants. Although Krejcie and Morgan's (1970) sampling framework assumes simple random sampling, the final sample size exceeded the recommended minimum, which helped mitigate potential reductions in statistical power associated with cluster‐based sampling using intact classes. The descriptive statistics for participants’ age and proficiency level in both groups are presented in Table 1.

**TABLE 1:** Central indices and dispersion of respondents' age and level.

	Experimental group				Control group			
	Mean	Std. deviation	Min	Max	Mean	Std. deviation	Min	Max
Age	15.99	1.79	14	20	15.94	1.73	14	20
Level	36.86	4.59	31	45	35.76	4.13	31	45

The frequency and percentage distribution of participants’ gender in the experimental and control groups are reported in Table 2.

**TABLE 2:** Frequency and percentage of the gender variable.

		Experimental group		Control group	
		Frequency	Percentage (%)	Frequency	Percentage (%)
Gender	Male	49	49.0	51	50.0
	Female	51	51.0	49	50.0
	Total	100	100.0	100	100.0

### 3.3 Instruments

All three instruments were standardized, previously validated questionnaires that were used in their original form without any modification. They have been widely employed in prior research and demonstrate strong psychometric properties.

### 3.4 Academic Development Questionnaire

Learners’ academic development was measured using a standardized academic development questionnaire designed to assess participants’ perceived progress in core English language skills, including vocabulary growth, comprehension, grammatical accuracy, and overall language performance. The conceptualization of academic development was grounded in sociocognitive perspectives on language learning that emphasize sustained academic growth rather than isolated test scores (Wells 2010).

The questionnaire is a previously validated instrument that was administered in its original form without modification. In the Iranian EFL context, academic development and language achievement have frequently been examined in relation to affective and cognitive classroom variables (e.g., Pishghadam et al. 2013; Shabani and Gholami 2020), which supports the relevance of this construct for the present study. Pilot testing with learners from a comparable population yielded a Cronbach's alpha of 0.87, indicating strong internal consistency reliability.

### 3.5 Cognitive Engagement Questionnaire

Cognitive engagement was assessed using a standardized questionnaire that measures learners’ mental effort, sustained attention, and persistence during learning activities. The instrument is grounded in well‐established models of learner engagement, particularly the conceptualization of cognitive engagement as active mental investment in learning tasks (Fredricks et al. 2004; Reeve 2013).

The questionnaire was administered in its original validated form without any modification to the items or structure. Engagement‐related constructs have been widely examined in Iranian EFL research, particularly in relation to motivational and affective classroom variables (e.g., Shabani and Gholami 2020), highlighting the relevance of cognitive engagement for language learning contexts. Pilot testing with a similar group of Iranian EFL learners indicated good reliability, with a Cronbach's alpha of 0.89.

### 3.6 Motivation for Learning Questionnaire

Learners’ motivation for learning English was measured using a standardized motivation for learning questionnaire grounded in SDT, which conceptualizes motivation in terms of intrinsic and extrinsic regulatory processes (Ryan and Deci 2017, 2020). The instrument assesses learners’ interest, enjoyment, effort, and attitudes toward learning English.

This questionnaire is a validated instrument that was used in its original form without modification. Motivation has been extensively examined in the Iranian EFL context, with studies consistently demonstrating its strong association with engagement and language achievement (e.g., Khajavy and Ghonsooly 2017; Pishghadam et al. 2013). Pilot testing in the present context yielded excellent internal consistency reliability (Cronbach's alpha = 0.91).

## 4 Intervention and Procedure

### 4.1 Experimental Group

Participants in the experimental group received song‐based English instruction over an 11‐week period. Each instructional week focused on one English song selected for its age‐appropriate content, high‐frequency vocabulary, and simple grammatical structures suitable for elementary‐level EFL learners. The selection of songs was guided by pedagogical considerations common in EFL classrooms and by prior research emphasizing the value of rhythm, repetition, and familiarity in language learning.

The songs used during the intervention included

1. Colors and Shapes
2. Teddy Bear
3. Head and Shoulders
4. Feelings
5. Fruits
6. Uncle Paul
7. Goodbye
8. Ali Baba's Farm
9. This Is the Way
10. Days of the Week

These songs are widely used in EFL instruction and are particularly suitable for learners with limited proficiency, as they provide repeated exposure to basic vocabulary and formulaic language.

Each lesson followed a three‐phase instructional structure, consistent with communicative and task‐based language teaching principles:

1. Pre‐listening activities, including vocabulary pre‐teaching, visual support, and prediction tasks, designed to activate background knowledge and facilitate comprehension.
2. While‐listening activities, such as singing along, clapping or moving to rhythmic patterns, and lyric gap‐fill tasks, aimed at enhancing phonological awareness, attention, and engagement.
3. Post‐listening activities, including role‐play, lyric rewriting, short discussions, or guided oral practice, intended to promote meaningful language use and productive skills.

This instructional sequence ensured that learners were exposed to both receptive and productive language practice and aligns with principles of multimodal learning, which emphasize the integration of auditory, verbal, and kinesthetic channels to enhance attention and memory (Mayer 2009; Moreno et al. 2011). The inclusion of singing and interaction‐based tasks also reflects recommendations from second language acquisition research highlighting the importance of meaningful input, output, and learner engagement.

In the Iranian EFL context, where instruction is often dominated by textbook‐based and form‐focused practices, song‐based activities have been suggested as a way to increase learner motivation and classroom engagement (Pishghadam et al. 2013). The present intervention was designed to address these contextual characteristics while remaining consistent with institutional curricular requirements.

### 4.2 Control Group

Participants in the control group studied the same instructional content over the same 11‐week period using traditional instructional methods, including teacher explanations, textbook exercises, repetition drills, and question–answer practice. No songs or musical materials were used in the control condition. Instructional time, learning objectives, and target language items were kept identical across groups to ensure that any observed differences in outcomes could be attributed to the presence or absence of song‐based instruction rather than to content coverage or time‐on‐task.

### 4.3 Instructional Fidelity

To minimize potential researcher bias and ensure consistency in implementation, the intervention was conducted by a qualified EFL instructor who was not a member of the research team. The instructor received standardized lesson plans for both experimental and control conditions prior to the intervention. Throughout the instructional period, periodic classroom observations were conducted to confirm adherence to the prescribed procedures and to verify that instructional differences between groups were limited to the use of songs in the experimental condition. These procedures were implemented to strengthen internal validity and to ensure fidelity of treatment across instructional settings.

### 4.4 Data Collection

Data were collected at three time points: pretest, immediate posttest, and follow‐up. The pretest was administered prior to the commencement of the instructional intervention to establish baseline equivalence between groups. The immediate posttest was administered at the conclusion of the 11‐week instructional period to assess short‐term instructional effects. The follow‐up test was administered 4 weeks after the completion of the intervention to examine the sustainability of observed effects. At each measurement point, participants completed the academic development questionnaire, cognitive engagement questionnaire, and motivation for learning questionnaire. The same instruments and administration procedures were used across all three time points to ensure consistency and comparability of scores.

All questionnaires were administered during regular class sessions under standardized conditions. Instructions were provided orally and clarified in learners’ first language where necessary to ensure comprehension. Participants were informed that their responses would remain confidential and would not affect their course grades. To minimize testing effects, no feedback was provided on questionnaire responses, and no additional instructional activities related to the measurement instruments were introduced between testing occasions. The use of repeated measurements enabled the examination of within‐group changes over time and between‐group differences across the three measurement points, consistent with the repeated‐measures analytical procedures employed in the study.

### 4.5 Intervention Period

Following the pretest administration, the experimental group received song‐based instruction, while the control group received traditional instruction, over an 11‐week period. No data were collected during the intervention to avoid disrupting instructional routines and to minimize testing effects. Instructional fidelity was monitored through standardized lesson plans and periodic observations, ensuring consistent implementation across groups.

### 4.6 Posttest Administration

At the conclusion of the intervention period, the same three questionnaires were readministered to both groups as posttests. The posttest administration procedures mirrored those used during the pretest phase in terms of timing, instructions, and classroom conditions to maintain procedural equivalence. The use of identical instruments at both measurement points allowed for direct comparison of pretest and posttest scores and supported the internal validity of the study.

### 4.7 Ethical Considerations in Data Collection

All data collection procedures adhered to established ethical guidelines for research involving human participants. Participation was voluntary, informed consent was obtained from all adult learners, and parental consent was secured for participants under the age of 18. Participants were assured of confidentiality and anonymity, and all collected data were coded and stored securely for research purposes only (Dörnyei 2007; Mackey and Gass 2021).

### 4.8 Data Analysis

The collected data were analyzed using SPSS version 28, following a systematic analytic plan aligned with the research questions and the study's quasi‐experimental design. Both descriptive and inferential statistical procedures were employed to examine the effects of song‐based instruction on learners’ academic development, cognitive engagement, and motivation for learning.

First, descriptive statistics, including means, standard deviations, minimums, and maximums, were computed to summarize participants’ demographic characteristics and overall performance at the pretest, posttest, and follow‐up stages. Reliability analyses were conducted for each questionnaire using Cronbach's alpha coefficients, confirming acceptable to high internal consistency for all instruments in the main study.

Second, assumption testing was performed to ensure the suitability of parametric analyses. Normality of score distributions was examined using the Kolmogorov–Smirnov test, and homogeneity of variances was assessed using Levene's test at each measurement point. To evaluate the assumption of sphericity required for repeated‐measures analyses, Mauchly's test of sphericity was conducted for each dependent variable. Where the sphericity assumption was violated, Greenhouse–Geisser corrections were applied, consistent with recommended statistical practice.

Third, independent‐samples t‐tests were conducted to compare the experimental and control groups at the pretest stage in order to confirm initial group equivalence. Additional independent‐samples t‐tests were used to compare group means at the posttest and follow‐up stages, providing an initial indication of group differences over time.

To examine changes across time and to evaluate the effects of the instructional intervention more rigorously, repeated‐measures analysis of variance (ANOVA) was employed for each dependent variable, with time (pretest, posttest, follow‐up) as the within‐subjects factor and group (experimental vs. control) as the between‐subjects factor. The primary focus of these analyses was the time × group interaction, which indicates whether changes over time differed significantly between the two instructional conditions.

Finally, effect sizes were reported to assess the magnitude and practical significance of the observed effects. Partial eta squared (*η* ^2^) values were reported for repeated‐measures ANOVA results, along with observed power statistics, in accordance with APA reporting standards. The reporting of effect sizes was intended to complement significance testing and to provide a more meaningful interpretation of the instructional impact. By integrating descriptive statistics, assumption testing, independent‐samples comparisons, and repeated‐measures ANOVA, the data analysis procedures provided a comprehensive and methodologically rigorous evaluation of the effects of song‐based instruction on EFL learners’ academic development, cognitive engagement, and motivation for learning.

## 5 Results

### 5.1 Descriptive Statistics

This section presents the results of the statistical analyses conducted to examine the effects of song‐based instruction on EFL learners’ academic development, cognitive engagement, and motivation for learning. The results are reported in two stages: first, descriptive statistics are presented to provide an overview of group‐level trends, followed by inferential analyses to test the research hypotheses. Descriptive statistics for academic development, cognitive engagement, and motivation across the pretest, posttest, and follow‐up stages are summarized in Table 3.

**TABLE 3:** Means (SD), minima, and maxima for pretest, posttest, and follow‐up; independent‐samples *t*‐tests.

Period	Variable		Experimental group				Control group				*t* test	
		*N*	Mean	Std. deviation	Min	Max	Mean	Std. deviation	Min	Max	*t* value	*p* value
**Pretest**	Academic development	100	116.67	11.16	89	149	117.41	10.19	96	141	−0.490	0.625
	Cognitive engagement	100	48.83	8.15	40	87	47.84	7.51	34	72	0.894	0.373
	Motivation for learning	100	55.03	8.60	43	93	54.00	7.60	37	69	0.897	0.371
**Posttest**	Academic development	100	130.65	9.53	119	159	125.87	10.13	101	150	3.437	0.001
	Cognitive engagement	100	53.31	8.24	42	87	47.27	7.67	31	74	5.364	0.001
	Motivation for learning	100	62.10	6.94	44	80	58.37	7.64	42	69	3.613	0.001
**Delaye**d **test**	Academic development	100	127.10	7.83	108	146	123.91	9.53	101	143	2.587	0.010
	Cognitive engagement	100	52.78	8.42	41	87	46.92	7.90	31	74	5.076	0.001
	Motivation for learning	100	61.84	6.78	44	78	58.07	7.67	41	69	3.683	0.001

## 6 Inferential Analysis

### 6.1 Assumption Checks for Repeated‐Measures ANOVA

Normality (Kolmogorov–Smirnov) and homogeneity of variances (Levene's) were evaluated separately for each time point and group (see Table 4). Results supported normality and equality of variances across groups. Mauchly's test of sphericity (see Table 5) was statistically significant for all three outcomes, so Greenhouse–Geisser corrections were applied in the repeated‐measures analyses.

**TABLE 4:** Kolmogorov–Smirnov normality tests and Levene's test of homogeneity.

		Normality test (K–S)				Variance equality Levene test			
		Experimental group		Control group					
Variable	Period	Test statistic	*p* value	Test statistic	*p* value	Levene statistic	df1	df2	*p* value
**Academic development**	Pretest	0.05	0.200^*^	0.06	0.200^*^	1.38	1	198	0.241
	Posttest	0.07	0.200^*^	0.06	0.200^*^	0.13	1	198	0.719
	Delayed test	0.06	0.200^*^	0.06	0.200^*^	0.79	1	198	0.377
**Cognitive engagement**	Pretest	0.07	0.200^*^	0.07	0.200^*^	0.05	1	198	0.830
	Posttest	0.07	0.195	0.07	0.200^*^	0.00	1	198	0.982
	Delayed test	0.07	0.200^*^	0.08	0.084	0.18	1	198	0.674
**Motivation for learning**	Pretest	0.05	0.200^*^	0.08	0.080	0.13	1	198	0.724
	Posttest	0.08	0.140	0.08	0.162	0.01	1	198	0.915
	Delayed test	0.07	0.185	0.08	0.110	0.78	1	198	0.379

* No significant.

**TABLE 5:** Mauchly's test of sphericity and correction decision.

Within‐subjects effect	Mauchly's *W*	Chi‐square	df	*p* value	Conclusion
Academic development	0.16	367.26	2	0.001	Greenhouse–Geisser
Cognitive engagement	0.09	464.90	2	0.001	Greenhouse–Geisser
Motivation for learning	0.02	741.80	2	0.001	Greenhouse–Geisser

### 6.2 Repeated‐Measures ANOVA Results

Table 6 displays the repeated‐measures ANOVA outcomes for each dependent variable, including between‐group effects, within‐subjects (time) effects under the Greenhouse–Geisser correction, and the time × group interaction, together with partial eta squared and observed power.

**TABLE 6:** Repeated‐measures ANOVA (Greenhouse–Geisser where applicable).

		Mean		Test							
		Experimental group	Control group		Type III sum of squares	df	Mean square	*F*	*p* value	Partial eta squared	Power
**Academic development**	Pretest	116.67	117.41	Between group	871.01	1.00	871.01	4.95	0.027	0.02	0.60
	Posttest	130.65	125.87	Within group (time)	13675.93	1.08	12615.97	123.44	0.001	0.38	1.00
	Follow	127.10	123.91	Within group (time × group)	807.46	1.08	744.88	7.29	0.006	0.04	0.79
**Cognitive engagement**	Pretest	48.83	47.84	Between group	2769.20	1	2769.20	22.72	0.001	0.10	1.00
	Posttest	53.31	47.27	Within group (time)	420.72	1.05	400.86	6.05	0.014	0.03	0.70
	Follow	52.78	46.92	Within group (time × group)	820.86	1.05	782.11	11.81	0.001	0.06	0.94
**Motivation for learning**	Pretest	55.03	54.00	Between group	1212.68	1	1212.68	10.15	0.002	0.05	0.89
	Posttest	62.10	58.37	Within group (time)	4159.36	1.01	4111.20	79.91	0.000	0.29	1.00
	Follow	61.84	58.07	Within group (time ×group)	246.65	1.01	243.80	4.74	0.030	0.02	0.59

## 7 Hypotheses Testing

### 7.1 Hypothesis 1

Learning language through songs has a significant effect on academic development for EFL learners. Consistent with Table 6, the between‐groups effect on academic development was significant (*F* = 4.95, *p* = 0.027, partial *η* ^2^ = 0.02). The within‐subjects effect of time was also significant (*F* = 123.44, *p* = 0.001, partial *η* ^2^ = 0.38), indicating changes from pretest to posttest and follow‐up. The time × group interaction reached significance (*F* = 7.29, *p* = 0.006, partial *η* ^2^ = 0.04), reflecting greater gains over time for the experimental group.

### 7.2 Hypothesis 2

Learning language through songs has a significant effect on cognitive engagement for EFL learners. As shown in Table 6, the between‐groups effect was significant (*F* = 22.72, *p* = 0.001, partial *η* ^2^ = 0.10). The within‐subjects (time) effect was significant (*F* = 6.05, *p* = 0.014, partial *η* ^2^ = 0.03). The time × group interaction was significant as well (*F* = 11.81, *p* = 0.001, partial *η* ^2^ = 0.06), indicating that engagement increased more in the experimental group across occasions.

### 7.3 Hypothesis 3

Learning language through songs has a significant effect on motivation for learning for EFL learners. The between‐groups effect was significant (*F* = 10.15, *p* = 0.002, partial *η* ^2^ = 0.05). The within‐subjects (time) effect was significant (*F* = 79.91, *p* < 0.001, partial *η* ^2^ = 0.29). The time × group interaction was significant (*F* = 4.74, *p* = 0.030, partial *η* ^2^ = 0.02), showing greater longitudinal improvement for the experimental group. Overall, the descriptive and inferential results converge to indicate that the song‐based instruction produced meaningful improvements in learners’ academic development, cognitive engagement, and motivation relative to the control group.

Even though the reported effect sizes ranged from small to moderate, results of this scale are typical in classroom‐based educational research and can still hold practical significance when the effects continue over time and occur within normal teaching setting. The maintenance of these effects at the follow‐up stage further strengthens their pedagogical significance.

## 8 Discussion

This study examined the immediate and sustained effects of song‐based instruction on EFL learners’ academic development, cognitive engagement, and motivation for learning. Using a quasi‐experimental design with pretest, posttest, and follow‐up measurements, the findings demonstrated that learners exposed to song‐based instruction outperformed those receiving traditional instruction across all three outcome variables. Importantly, the observed gains were not limited to the immediate post‐intervention stage but were largely maintained at the follow‐up, indicating the durability of the instructional effects. These results underscore the pedagogical value of integrating songs into EFL instruction as a means of enhancing both cognitive and affective dimensions of language learning.

### 8.1 Discussion of Hypothesis 1: Academic Development

The first hypothesis examined whether learning English through songs leads to greater academic development compared to traditional instructional approaches. The findings supported this hypothesis, revealing that learners in the song‐based instruction group demonstrated significantly higher academic development, with gains maintained at the follow‐up stage. This suggests that song‐based instruction contributes not only to immediate improvement but also to sustained academic growth.

These findings align with theoretical perspectives emphasizing the role of meaningful, repeated input in language development. The rhythmic and repetitive nature of songs may facilitate phonological processing and memory consolidation, supporting learners’ comprehension and vocabulary development over time (Schön et al. 2004; Besson et al. 2011). From a sociocognitive perspective, songs also provide emotionally engaging input, which may promote deeper processing and longer‐term retention (Krashen 1985; Wells 2010).

The sustained nature of the observed gains is particularly noteworthy in light of recent evidence syntheses highlighting the lack of delayed measurements in much song‐based research (Hamilton et al. 2024). By demonstrating maintained academic development at follow‐up, the present study extends previous findings that have focused primarily on short‐term linguistic outcomes and contributes to a more comprehensive understanding of how songs support academic development in EFL contexts.

### 8.2 Discussion of Hypothesis 2: Cognitive Engagement

The second hypothesis investigated whether song‐based instruction enhances learners’ cognitive engagement, operationalized through attention, retention, and participation. The results confirmed that learners exposed to songs exhibited higher levels of cognitive engagement than those in traditional classrooms, with these effects persisting at the follow‐up stage.

This finding is consistent with theoretical accounts of cognitive engagement, which emphasize sustained mental effort and active involvement as key determinants of learning quality (Fredricks et al. 2004; Reeve 2013). Multimodal learning theory provides a strong explanatory framework for this effect, suggesting that integrating auditory and verbal channels enhances attentional focus and memory formation (Mayer 2009). Neurocognitive research further supports this view, demonstrating that musical input activates neural systems associated with attention and working memory more extensively than speech‐only input (Patel 2011; Moreno et al. 2011).

The maintenance of cognitive engagement at the follow‐up stage suggests that song‐based instruction may foster learning habits characterized by sustained attentional control and persistence, rather than momentary engagement driven solely by novelty. This finding is particularly relevant in EFL contexts, where maintaining learner attention and participation over time is a persistent instructional challenge. The persistence of these engagement gains at the follow‐up stage suggests that the observed effects cannot be attributed solely to short‐term novelty, but rather reflect more durable changes in learners’ cognitive investment.

### 8.3 Discussion of Hypothesis 3: Motivation for Learning

The third hypothesis examined whether song‐based instruction enhances learner motivation and attitudes toward learning English. The results supported this hypothesis, showing that learners in the experimental group reported higher levels of motivation than those in the control group, with these motivational gains remaining stable at the follow‐up stage.

These findings are well aligned with SDT, which posits that learning environments supporting autonomy, competence, and relatedness foster more self‐determined forms of motivation (Ryan and Deci 2017, 2020). Songs may contribute to such environments by reducing anxiety, increasing enjoyment, and promoting shared classroom experiences, thereby strengthening learners’ emotional connection to the language (Dörnyei and Ushioda 2013; Lamb 2017).

The sustained motivational effects observed in the present study extend previous research that has primarily documented short‐term increases in enjoyment or positive attitudes associated with music‐based activities. By demonstrating that motivational benefits persist beyond the immediate instructional period, the findings suggest that song‐based instruction may contribute to longer‐term motivational orientations that support continued engagement and learning.

Taken together, the findings support an integrated explanatory model in which song‐based instruction enhances motivation, which in turn promotes deeper cognitive engagement and ultimately supports academic development over time. This model aligns with sociocultural and sociodynamic perspectives on language learning, which emphasize the interdependence of affective, cognitive, and social processes (Vygotsky 1978). From a pedagogical standpoint, the results suggest that songs should not be viewed merely as supplementary or motivational tools but rather as systematic instructional resources capable of supporting sustained learning outcomes. Incorporating songs into EFL curricula may be particularly beneficial in contexts where learners experience anxiety, low motivation, or limited opportunities for authentic language use. Beyond demonstrating effectiveness, the findings contribute to theory by illustrating how multimodal, affectively rich instruction can simultaneously support motivational, cognitive, and academic processes over time in EFL learning contexts. Although multimodal learning theory (Mayer 2009) and neurocognitive research (Patel 2011; Moreno et al. 2011) provide a theoretical basis for expecting enhanced processing under music‐supported instruction, the present findings extend these accounts in important ways. Rather than attributing the observed gains solely to multimodal input or neural activation mechanisms, the results suggest that the effectiveness of song‐based instruction may stem from the interaction between multimodal processing, sustained cognitive engagement, and motivational regulation. The durability of the effects observed at the 4‐week follow‐up further indicates that songs may function not only as perceptual enhancers but also as socio‐affective mediators that promote continued engagement with linguistic input. In this sense, the study advances an integrated explanation that connects cognitive processing advantages with motivational and sociocultural dynamics in classroom‐based EFL learning.

## 9 Conclusion

This study demonstrated that song‐based instruction has significant immediate and sustained positive effects on EFL learners’ academic development, cognitive engagement, and motivation for learning. Learners exposed to songs consistently outperformed those receiving traditional instruction, and these gains were largely maintained at the follow‐up stage. The results indicate that songs contribute beyond simple motivational support, creating a learning context that combines multiple modes and emotional engagement, which may encourage greater cognitive involvement and support sustained academic development.

Grounded in established motivational and learning theories, the results highlight the pedagogical value of integrating songs systematically into EFL instruction, particularly in contexts where learner engagement and motivation are ongoing challenges. Overall, the study contributes empirical evidence for the durability of song‐based learning effects and supports its inclusion as a meaningful instructional approach in EFL classrooms.

### 9.1 Pedagogical Implications

The findings have clear implications for EFL pedagogy. For teachers, incorporating songs into regular classroom practice offers a practical strategy for increasing learner engagement, supporting sustained attention, and fostering positive motivational orientations toward language learning. Song‐based activities can be integrated into existing curricula to reinforce linguistic input while simultaneously addressing learners’ affective needs. For curriculum designers and policymakers, the results suggest that music‐integrated materials may enhance learner outcomes and should be considered as part of communicative and multimodal language programs, particularly in contexts where motivation and engagement present ongoing challenges.

In the context of Iranian EFL education, which often relies on structured curricula and teacher‐centered instructional practices, song‐based instruction may serve as a practical supplementary strategy for increasing learner engagement and motivation. Teachers can incorporate songs aligned with lesson themes to reinforce vocabulary, pronunciation, and listening comprehension while maintaining alignment with mandated textbooks and learning objectives. Short song‐based activities—such as lyric gap‐fills, vocabulary identification, and pronunciation practice—can be integrated into regular lessons without requiring substantial curricular changes. Moreover, the use of music may help create a more supportive and engaging classroom environment, potentially encouraging greater learner participation and sustained attention. Given the growing access to digital resources in Iranian educational settings, teachers may also use multimedia platforms to introduce authentic English songs that complement classroom instruction while remaining culturally appropriate.

### 9.2 Limitations and Directions for Future Research

Despite its contributions, the study has several limitations. The sample was drawn from a specific instructional context, which may limit the generalizability of the findings. Although the inclusion of a follow‐up measurement strengthened the research design, the intervention and follow‐up period were relatively short. In addition, reliance on self‐report instruments may have introduced response bias, and learners’ familiarity with selected songs may have influenced levels of engagement and motivation. In this study, academic development refers to learners’ perceived academic growth rather than objective achievement outcomes.

Future research should extend this work by examining song‐based instruction over longer instructional periods and follow‐up intervals, and across more diverse learner populations and proficiency levels. Employing longitudinal and mixed‐methods designs, as well as behavioral or neurocognitive measures of engagement, could provide deeper insight into the mechanisms underlying sustained learning effects. Further investigation into the impact of different musical genres, cultural relevance, and digital music‐based tools would also help refine the pedagogical application of song‐based language learning. Nevertheless, perceived academic development remains a theoretically meaningful construct, as learners’ self‐assessments are closely linked to motivation, engagement, persistence, and long‐term learning trajectories in second language acquisition.

## Author Contributions

**Yanyi Wang**: methodology, validation, writing – review and editing, conceptualization, investigation. **Morteza Jabrealy**: conceptualization, investigation, writing – original draft, validation, data curation. **Siros Izadpanah**: conceptualization, writing – original draft, writing – review and editing, validation, methodology, project administration, supervision.

## Funding

The authors have nothing to report.

## Ethics Statement

Ethical approval for this study was obtained from the Research Ethics Committee of Islamic Azad University–Zanjan Branch (approval ID: IR.IAU.Z.REC.1404.057). All procedures were conducted in accordance with the ethical principles of the Declaration of Helsinki. Participation was voluntary, informed consent was obtained from all participants prior to data collection, and participants were informed of their right to withdraw at any time without penalty. Anonymity and confidentiality of all data were strictly maintained throughout the research process.

## Consent

Written informed consent was obtained from all subjects before the study. All the participants filled out consent forms.

## Conflicts of Interest

The authors declare no conflicts of interest.

## Data Availability

The data will be available upon request to the corresponding author.
